# Interactions Between the Neuroendocrine System and T Lymphocytes in Diabetes

**DOI:** 10.3389/fendo.2018.00229

**Published:** 2018-05-17

**Authors:** Luz Andreone, María Laura Gimeno, Marcelo J. Perone

**Affiliations:** Instituto de Investigación en Biomedicina de Buenos Aires (IBioBA) – CONICET – Instituto Partner de la Sociedad Max Planck, Buenos Aires, Argentina

**Keywords:** T lymphocytes, inflammation, insulin resistance, adipose, muscle, liver, cytokines

## Abstract

It is well established that there is a fine-tuned bidirectional communication between the immune and neuroendocrine tissues in maintaining homeostasis. Several types of immune cells, hormones, and neurotransmitters of different chemical nature are involved as communicators between organs. Apart of being key players of the adaptive arm of the immune system, it has been recently described that T lymphocytes are involved in the modulation of metabolism of several tissues in health and disease. Diabetes may result mainly from lack of insulin production (type 1 diabetes) or insufficient insulin and insulin resistance (type 2 diabetes), both influenced by genetic and environmental components. Herein, we discuss accumulating data regarding the role of the adaptive arm of the immune system in the pathogenesis of diabetes; including the action of several hormones and neurotransmitters influencing on central and peripheral T lymphocytes development and maturation, particularly under the metabolic burden triggered by diabetes. In addition, we comment on the role of T-effector lymphocytes in adipose and liver tissues during diabetes, which together enhances pancreatic β-cell stress aggravating the disease.

## Introduction

Pioneering work in the 1980s provided the first evidence of the cross-talk between the neuroendocrine and immune systems (1–4). It is now well established that there is a fine-tuned bidirectional communication between these tissues in maintaining homeostasis. Several types of immune cells, hormones, and neurotransmitters of different chemical nature are involved as communicators between organs influencing immune development and function (5, 6). Additionally, it has been described that T lymphocytes apart of being key players of the adaptive arm of the immune system, are involved in the modulation of metabolism in several tissues in health and disease (7–13).

Diabetes is a highly prevalent endocrine-metabolic disease with a constant growing rate, affecting nearly half a billion people worldwide (14). It is characterized by an imbalance in glucose homeostasis, which result mainly from lack of insulin production in the pancreas [type 1 diabetes (T1D)] or insufficient insulin production and peripheral insulin resistance [type 2 diabetes (T2D)] both influenced by genetic and environmental components.

In this Review, we discuss existing data about the role of the adaptive arm of the immune system in the diabetes pathophysiology; including the action of several hormones and neurotransmitters influencing on central and peripheral T lymphocytes development and maturation, particularly under the metabolic burden triggered by diabetes. In addition, we comment on the role of T-effector lymphocytes in peripheral tissues during diabetes, which together enhance pancreatic β-cell stress aggravating the disease.

## The Role of T Cells in the Pathogenesis of T1D

Type 1 diabetes is a T cell-mediated autoimmune disease that selectively destroys insulin-producing β-cells. The key roles for both CD4^+^ and CD8^+^ T cells in the immune response that drives T1D have been extensively described (15, 16). It is now widely accepted that endogenous and/or exogenous initiating factors, operating on a genetic susceptibility background and permissive environmental framework, are necessary for the development of autoreactive T lymphocytes that infiltrate pancreatic islets (insulitis) (17).

While the association of class II HLA genes polymorphisms with T1D risk has been known for over 40 years (18), recent single-nucleotide polymorphisms (SNPs) genotyping technologies allow the description of many additional T1D susceptibility genes (19–21). Intriguingly, most of these genes are coding for cytokines, cytokine receptors, and factors that regulate T cell differentiation, suggesting that control of T cell identity may be an important element of the genetic contribution to disease susceptibility and onset.

The process of T cell differentiation that takes place in the thymus is regulated by many molecules such as hormones, neuropeptides, and neurotransmitters involving both endocrine and paracrine signaling pathways (6). A variety of peptide and nonpeptide hormones modulate the proliferation, differentiation, migration, and apoptosis of developing thymocytes. The dysfunction in the hormonal control of T cell differentiation is associated with the development of diseases that are influenced by immune cells, including diabetes.

Currently, there is a wide consensus that T1D is a Th1-mediated pathology and INF-γ is implicated as the main driver cytokine of the process of autoimmune islet destruction; meanwhile, Th2 cell-type would play a protective role (22–29). However, not all emerging data from mouse models and patients are consistent with the dominance of a Th1 response in T1D; multiple additional T cell differentiation phenotypes are now recognized with distinct functions (30, 31).

The role of Th17 lymphocytes in T1D is not fully understood. Murine models and human studies suggest that IL-17 is upregulated in the early stages of diabetes development but it is still not clear if this cytokine, or indeed if the Th17 subset, is necessary for disease (32–38). It was shown that genetic IL-17 silencing had no effect and did not protect NOD mice from spontaneous autoimmune diabetes (39). Some studies suggested that an increase of T cells co-expressing IFN-γ and IL-17 could be a feature of T1D development (36, 40–42). Several types of cells of the immune system, attracted by signals from the islets, contribute to the selective β-cell death through the release of cytotoxic inflammatory cytokines, such as IL-1β, IFN-γ, and TNF-α (43, 44). Recent studies performed in human β-cells suggested that pancreatic IL-17 contributes to the pathogenesis of T1D by two mechanisms, exacerbating β-cell apoptosis and increasing local production of chemokines by islets exposed to pro-inflammatory cytokines (e.g., IL-1β + IFN-γ and TNF-α + IFN-γ) (45). In a study of children in various phases of diabetes-associated autoimmunity and clinical disease upregulation of IL-17 and Th1/Th17 plasticity in peripheral blood were observed in stages of advanced β-cell autoimmunity and impaired glucose tolerance and clinical T1D (42). Activated Th17 immunity was not observed in patients with early β-cell autoimmunity, indicating that Th17 may be a marker of late preclinical autoimmune diabetes which correlates with impaired β-cell function. Analysis of pancreatic lymph nodes in T1D patients showed higher frequency of Th17 cells in comparison with non-diabetic controls (46). The consolidation of Th17 cells as part of T1D pathophysiology focused attention on additional cytokines, outside of those associated classically with the Th1/Th2 paradigm (IFN-γ and IL-4, respectively).

IL-21 is a pleiotropic cytokine produced mainly by T follicular helper (Tfh) cells, Th17 cells, and natural killer (NK) cells. Although it has been demonstrated that IL-21 enhances Th17 differentiation and it can be produced by Th17 cells to exert autocrine feedback (47, 48), existing data indicated that the role of IL-21 in the development of diabetes is more than just an effect on Th17 differentiation. Preclinical studies performed in the NOD mice demonstrate that the IL-21 pathway is critical for disease development (49–51). It acts in a paracrine and autocrine fashion affecting the differentiation and function of several immune cell types in the context of T1D, including CD4^+^ and CD8^+^ T cells, NK cells, B cells, macrophages, and dendritic cells (52, 53). Moreover, transgenic overexpression of IL-21 in the pancreatic islets results in autoreactive T cell infiltration and β-cell apoptosis in C57BL/6 mice, a strain free of any kind of autoimmunity signs (54).

As aforementioned, IL-21 is the signature cytokine for Tfh cells, the T lymphocyte subset that is specialized in providing help for B cell antibody production (55). Islet autoantibodies are the best currently available biomarkers to detect ongoing autoimmune process and T1D development risk (56). The production of such antibodies by autoreactive B cells is largely dependent on the function of Tfh cells. By means of an unbiased microarray approach and flow cytometry assay, a recent study assessed T cell differentiation in a mouse model of spontaneous autoimmune diabetes revealing that islet-specific T cells responding to pancreatic antigens show mainly the characteristic features of differentiated Tfh cells (57). Also, adoptive transfer of T cells with a Tfh phenotype from diabetic animals is highly efficient at inducing diabetes to murine recipients. Furthermore, peripheral memory CD4^+^ T cells from patients with T1D expressed elevated levels of Tfh cell markers (57). In accordance, an increase in peripheral blood Tfh cells has also been reported in three T1D patient independent cohorts, one of which comprised exclusively new-onset patients (58–60).

Interleukin-2 (IL-2) is critical for maintaining the function of the CD4^+^ regulatory T cells (Tregs), which in turn regulate autoreactive CD4^+^ effector T cells (Teffs) to prevent autoimmune diseases, such as T1D (61, 62). The involvement of the IL-2 pathway in the physiopathology of T1D first emerged from NOD mice; a reduced IL-2 production by the susceptibility allele (NOD disease-associated SNPs in IL-2 promoter) led to a consequent reduction of Treg function (63, 64). In humans, certain SNPs of the IL-2 receptor gene, *IL2RA*, encoding the α subunit (CD25) as well as of other genes in the IL-2 pathway, were identified as susceptibility determinants for T1D (65–68). Accordingly, an attenuated IL-2/IL-2R signaling was observed in Treg and Teff cells of T1D patients (69). In a clinical study with recently diagnosed T1D subjects, treatment with low doses of recombinant human IL-2 successfully induced a 10–20% increase in circulating Tregs whereas reduced Teffs, NK cells, and eosinophils (70); these findings lay the groundwork for the potential therapeutic use of rhIL-2 for treating T1D.

At present, emerging evidence suggests that pancreatic-resident Treg subsets have unique effects on the suppression of immune responses in T1D (71). Those distinguishable Treg subpopulations that reside in tissues exhibit special phenotype and function in response to local signals, thereby promoting tissue homeostasis (72). Among those special Treg subsets found in pancreatic tissues and pancreatic lymph nodes involved in preventing inflammation during T1D are: IL-10 secreting ICOS^+^ Tregs (73, 74), CXCR3^+^ Tregs (75), and TGF-β-expressing Tregs (76).

In summary, several studies regarding T cell differentiation in T1D clearly demonstrated not only the role of Th1 cells but also the possible involvement of other kind of T-effector cells co-expressing IFN-γ and IL-17, IL-21 producing T cells such as Tfh cells as well as circulating and pancreatic-resident Tregs.

## T Cells Contribution to Adipose Tissue Inflammation and Obesity-Associated Diabetes

Type 2 diabetes is a metabolic disease characterized by hyperglycemia resulting from either or both impaired β-cell insulin secretion and increased peripheral insulin resistance; particularly in muscle, liver, and fat (77). The pathogenesis of T2D is complex, it is a multifactorial disease that involves behavioral and environmental factors modulating T2D risk alleles in multiple genes. The pancreatic islets respond to the decrease in insulin-stimulated glucose uptake by enhancing their β-cell mass and insulin secretory activity. When β-cell function can no longer compensate for the prevailing insulin resistance, impaired glucose tolerance and T2D develop.

β-cell dysfunction precedes diabetes, and endoplasmic reticulum (ER) stress contributes to insulin secretory failure. β-cells are particularly susceptible to ER stress due to the high rate of insulin demand in response to rapid changes in glycemia levels. Many environmental factors, including inflammatory cytokines (78), reactive oxygen species (ROS) (79), and viral infections (80), may induce ER stress in β-cells associated with T1D triggering. Dysfunctional β-cells of NOD mice show feature ER stress before overt diabetes (81) and strategies directed to ameliorate ER stress may have therapeutic potential (82). Also, several lines of evidence link inflammation-associated obesity, ER stress, and T2D. The association of ER stress and T2D has been reviewed recently (83).

Inflammation was first linked to insulin resistance and T2D in the early 1990s; an induction of TNF-α expression was systemically and locally observed in adipose tissue from four different rodent models of obesity and diabetes (84). Since then, several studies have described elevated circulating levels of diverse inflammatory factors, such as acute-phase proteins, cytokines, and chemokines in patients with T2D (85–88). Currently, T2D is recognized as a chronic, low-grade inflammatory disease with involvement of pro-inflammatory cytokines and immune cells, including B and T cell subsets as pathogenic mediators (89, 90).

The inflammatory process observed in T2D is usually linked to obesity, a critical risk factor for the disease. Moreover, altered lipolysis in response to over nutrition and rapidly expanding adipose tissue results in elevation of pro-inflammatory saturated free fatty acids (FFAs). FFAs trigger metabolism-associated inflammation through toll-like receptors (TLRs), particularly TLR2 and TLR4, activating signaling pathways that lead to local adipose tissue infiltration by immune cells and systemic insulin resistance (91). The activation of TLR2/4 induces the production of inflammatory cytokines by dendritic cells, macrophages, endothelial cells, and pancreatic islets, as well. During diabetes, high circulating levels of glucose, FFAs, and pro-inflammatory cytokines contribute to insulin resistance and alterations in the immune system (91). Of note, the TLR2/TLR4 expression levels are upregulated in obese individuals (92). Moreover, TLR2- and TLR4-deficient mice are protected from the metabolic undesirable effects of high-fat diet (93) and experiments administering TLR2 antisense-oligonucleotides to high-fat-fed mice recovered insulin sensitivity in adipose tissue (94). Furthermore, nutrient excess may also induce local inflammation in the pancreatic islets (12, 95–97). Tissue inflammation has been detected in pancreatic islets of T2D patients, along with increased levels of cytokines and chemokines. Moreover, all T2D animal models investigated to date display some degree of insulitis (98, 99). TLR2/4 ligands are central in macrophages activation and consequent reduction of insulin secretion from pancreatic β-cells mainly by action of IL-1β and IL-6 on decreased insulin gene expression (100). Also, downstream MyD88-dependent and independent signaling pathways of FFAs-activated TLR2/4 induce differential gene expression and cellular responses leading to islet inflammation and β-cell dysfunction [reviewed in Ref. (101)].

Macrophages are the major immune cell type in adipose tissue, and its relative abundance increased from 5% in lean subjects to a level of up to 50% in obese patients. Moreover, the increase in number is accompanied by an evolution from the anti-inflammatory M2- to the pro-inflammatory M1-phenotype (102); adipose tissue macrophages (ATMs) produce a significant proportion of the inflammatory factors that are upregulated during obesity (95, 96, 103). Therefore, first studies on inflammatory regulation of T2D have been focused on the innate arm of the immune system. However, more recent studies suggest that adaptive immune cells, especially T lymphocytes, generally accumulate in obese adipose tissue in parallel with macrophages and also play a pivotal role in the pathophysiology of T2D (104). Moreover, studies in a mice model of T2D suggest that the accumulation of T lymphocytes in the adipose tissue might occur even before the arrival of macrophages (105).

T cells play a key role during the sequence of events that lead macrophage adipose tissue infiltration. In particular, CD8^+^ T cells are activated in adipose tissue which in turn, primer the recruitment and activation of macrophages within this tissue. In fact, infiltration of CD8^+^ effector (CD62L^−^ CD44^+^) T lymphocytes are described as one of the earliest events during the development of adipose tissue inflammation in mice due to obesity caused by *ad libitum* access to a high-fat diet (106). CD8^+^ T infiltration takes place in obese individuals too, as the expression of *CD8A* in subcutaneous adipose tissue was found elevated in comparison with lean subjects. Interestingly, CD8^+^ T lymphocytes not only precede adipose tissue infiltration by other immune cells, they are also required for the maintenance of inflammation in obese adipose tissue, since CD8^+^ T depletion attenuated adipose tissue inflammation and ATMs recruitment, and ameliorated insulin resistance and glucose intolerance in obese mice. CD8^−null^ mice fed a high-fat diet show moderate imbalance of glucose homeostasis. In this respect, gain of function experiments in where CD8^+^ T cells were administered into obese CD8^−null^ mice aggravate glucose intolerance and insulin resistance, reinforcing the notion that CD8^+^ T cells are essential for M1 macrophage infiltration and subsequent inflammation in diet-induced obese mice (106).

Visceral adipose tissue (VAT) inflammation involves a complex communication network between different T cell subpopulations expanded by factors that drive differentiation into several kinds of pro-inflammatory effectors. Adipose tissue T cell populations changed with increasing obesity in mice, and an increase in the ratio of CD8^+^ to CD4^+^ was reported by various research groups (9, 10, 106, 107). Particular T cell subpopulations play key roles in glucose homeostasis in human and mice. Winer and colleagues reported the importance of VAT resident CD4^+^ T lymphocytes as modulators of insulin sensitivity in mice under diet-induced obesity; glucose homeostasis was compromised when pathogenic IFN-γ-secreting Th1 cells accumulated in adipose tissue and overwhelmed the static numbers of Th2 and Treg cells. In fact, total absence of INF-γ improved insulin resistance in obese INF-γ KO mice in comparison with control animals having the same diet (108). It was reported that Rag1^−^ mice, known to be deficient in lymphocytes, developed a T2D phenotype on a high-fat diet, and when adoptively transferred with CD4^+^ T cells but not CD8^+^ T cells, normalized glucose tolerance; in particular Th2 signals from the transferred CD4^+^ T cells were crucial in the protective effect (10). Clinical studies have confirmed the abundant infiltrate of Th1, Th2, and Th17 CD4^+^ T cells, as well as IFN-γ_+_ CD8^+^ T cells in adipose tissue of healthy overweight and obese humans (109); pro-infammatory Th1, Th17, and IFN-γ_+_ CD8^+^ T cells were markedly increased in VAT relative to subcutaneus adipose tissue. Also, McLaughlin and colleagues confirmed the positive correlation between the relative dominance of Th1 vs Th2 responses in the adipose tissue and peripheral blood and insulin resistance.

A distinctive T cell subpopulation which infiltrates VAT, in a B-lymphocyte dependent way, has been recently identified and resembles senescence-T cells that show up in secondary lymphoid organs with age (110). Phenotypically they are distinguished by expression of CD44^hi^CD62L^lo^CD153^+^PD-1^+^ on the surface of CD4^+^ T cells and their feature characteristic is the large production of pro-inflammatory osteopontin upon T cell receptor (TCR) stimulation in parallel with compromised IFN-γ and IL-2 secretion. Moreover, they expressed increase senescence associated markers, such as β-gal, γ-H2AX, and *Cdkn1a/Cdkn2b*. This osteopontin-expressing T cells linked visceral adiposity with immune aging (110).

Invariant natural killer T (iNKT) cells are innate T cells involved in inflammatory responses. Adipose tissue-resident iNKT cells protect against obesity and metabolic disorder reducing inflammation in obese individuals (111); they are enriched in human adipose tissue and their number is reduced in obesity (112). iNKT cells express semi-invariant CD1d-restricted TCRs that recognize glycolipid antigens on major histocompatibility complex-like molecule CD1d (113, 114). Huh et al. reported that the absence of CD1d in adipocytes aggravates inflammation in adipose tissue and insulin resistance in obesity suggesting that adipose CD1d is a central activator of adipose iNKT cells. Activated iNKT cells would stimulate counter regulation of inflammation leading to reduced pro-inflammatory responses and insulin resistance in obesity (115).

The relationship between T2D and Th17 cells has also been studied (116). Obesity has been shown to promote expansion of peripheral or adipose tissue-resident IL-17-producing T cells, in human and mice models. In humans, peripheral Th17 cells are increase in T2D patients (117) and positively correlated with body mass index (BMI) but not in metabolically healthy obese subjects (118). Interestingly, T cells from obese T2D donors produced more IL-17 than that from non-diabetic counterparts and this production correlates with T2D severity (118). In diet-induced obese mice an IL-6-dependent expansion of the Th17 T cell pools was observed (119). Specific adipose tissue dendritic cells isolated from obese animals and humans were associated with the differentiation of Th17 cells *in vitro* (120). Studies performed by Zúñiga and colleagues showed an *in vitro* effect of IL-17 on differentiated adipocytes, impairing glucose uptake; *in vivo*, IL-17 deficiency enhanced glucose tolerance and insulin sensitivity in young mice (121).

The role of Treg cells in the maintenance of self-tolerance and the suppression of potentially autoreactive T cells is well known. However, the importance of Treg cells in metabolism has been recognized when it was found that lean adipose tissue enriched in Treg cells (~50% of the CD4^+^ T cell compartment) controls metabolic status. Indeed, Treg cells in adipose tissue of lean mice provide anti-inflammatory signals to prevent tissue inflammation. Interestingly, Treg cell proportion in the abdominal fat decreases dramatically with obesity (9, 10, 122) resulting in fat tissue inflammation and insulin resistance. Moreover, Feuerer et al. demonstrated that cytokines differentially synthesized by fat-resident Tregs directly affected the synthesis of inflammatory mediators and glucose uptake by cultured adipocytes. Winer et al. associated this Treg mediated protection to the production of IL-10 in ATMs and the restraint of pro-inflammatory macrophage activity, which improves insulin sensitivity.

In accordance, studies in humans showed that the relative proportion of Treg cells in visceral and subcutaneous fat decreased in patients with T2D and negatively correlated with BMI (9, 118) and that there is a decrease in Treg to Th17 and Th1 cell ratios (117). A recent study add complexity to the Treg role on the mechanisms underlying insulin resistance, supporting the concept that age-associated and obesity-associated IR are driven by distinct adipo-immune populations (123). Bapat and colleagues showed that a particular subset of fat-resident regulatory T cells (fTreg cells) accumulate in VAT as a function of age but not obesity. Additionally, the authors suggest that fTreg cells are functionally distinct from splenic Tregs; while certain canonical genes are similarly expressed, they have discrete expression signatures (i.e., higher expression levels of PPARγ and IL-33 receptor, ST2). Taking advantage of the high expression of ST2 on the surface of fTreg cells, Bapat and co-workers deplete fTreg cells by means of anti-ST2 administration. Interestingly, selective depletion of fTreg cells increases adipose tissue insulin sensitivity implicating these cells as drivers of age-associated insulin resistance (123). Contrary, *in vivo* stimulation of fTreg cells expansion within adipose tissue by treatment with IL-33 decreases insulin sensitivity. All these data suggest that distinct pathophysiologies undergo obesity and age-associated insulin resistance and support the notion that adipo-resident immune cells play a central role in adipose tissue glucose regulation and consequently, whole-body glucose homeostasis in mice.

Interestingly, recent evidences in mice and human suggested that the adipose tissue inflammation associated with obesity, in particular the T cell imbalance, and the impairment in insulin sensitivity, persist even after weight reduction (124, 125). It remains to be elucidated the precise mechanistic pathways of glucose regulation by T cells in human beings.

In summary, the evidence involving the role of T cells in adipose tissue inflammation and insulin resistance suggests that the interplay between T cells, macrophages, and adipocytes is essential. These cells communicate each other in the local adipose tissue environment to activate a sequence of events leading to an inflammatory state. It has been described the role of CD8^+^ T cells, Th1 and Th17 cells contributing to the obesity-induced insulin resistance phenotype, whereas Th2 cells and Tregs would play a protective role. However, the identity of the trigger that initiates T lymphocyte infiltration within adipose tissue in obesity still remains unknown.

## Liver and Gastrointestinal Resident T Cells in Metabolic Disorders

The liver participates in immunological responses and hepatocytes are also recognized as active immunological mediators among other well-known intrahepatic immune cells (126). There is a subset of innate-like T cells, named mucosal-associated invariant T (MAIT) cells, that recognizes small molecules presented on the non-polymorphic MHC-related protein 1 (MR1) by antigen-presenting cells and express a semi-invariant TCR (127). Like iNKT cells, these non-conventional T cells exhibit restricted TCR diversity recognizing metabolites on MR1 and play a major role in host protection from intracellular pathogens. MAIT cells are scarce in lymphoid tissues, comprising a high proportion of the total intrahepatic and gastrointestinal tract T cells population in humans, having a relevant role as an innate immune barrier against microbial invasion. However, their role in diseases begins to be clarified recently. Interestingly, MAIT cells activate under changes in the composition of gut microbiota and home to inflamed tissues. Magalhaes et al. reported for the first time the existence of MAIT cells abnormalities in severe obese and T2D patients (128). Both, obese and T2D patients showed a decreased in the number of circulating blood MAIT cells as well as dramatic changes in their functionality, i.e., an activated phenotype associated with high Th1- and Th17-type cytokines production. In obese individuals, an elevated number of MAIT cells in inflamed adipose tissue was found suggesting their recruitment from circulation.

Many studies have linked the microbiota, gut integrity, and metabolic disorders. MAIT cells might play a role involving the immune system as a fundamental part of these complex interactions. Recently, Rouxel et al. described that MAIT cells, exhibiting high production of granzyme B and pro-inflammatory cyokines, might directly kill β-cells in humans and NOD mice as well (129). As in the case for T2D patients, a reduced frequency of MAIT cells in peripheral blood of children with recent diagnosis of T1D was described, but not in those who are suffering from the disease for a long period of time. All these evidences highlight the role of MAIT cells in the maintenance of homeostasis within the complex interplay between mucosal integrity and normal islet responses. It would be interesting to investigate the functionality of gastric-resident MAIT cells in gastroparesis, a well-recognized complication of diabetes, since it has been demonstrated a connection between these cells with inflammatory bowel disease (130).

Although the mechanisms triggering and sustaining autoimmunity are not fully understood, the interaction of the intestinal environment with microbiota and, its epithelial integrity play a role in the development of T1D, and the disease in NOD mice (131, 132). A recent paper highlights the relevance of intestinal IL-10-producing type 1 regulatory T (Tr1) cells in the control of Teffs and development of diabetes (133). Increased differentiation of Tr1 cells may account by IL-27 and TGF-β action on intestine. These Tr1 cells have the ability to migrate to islets where they can suppress diabetogenic T cells *via* IL-10 signaling. Moreover, gut microbial metabolites augment the number and function of Treg cells, limiting the frequency of autoreactive T cells and protecting against autoimmune diabetes in NOD mice (134).

## Skeletal Muscle Resident T Cells and Glucose Homeostasis

Skeletal muscle is the predominant tissue of insulin-mediated glucose uptake in the postprandial state in humans (135); moreover, lipid accumulation in this tissue is associated with insulin resistance. Muscle insulin resistance is a major factor in the etiology of the metabolic syndrome and T2D (136). The increase in macrophages number within skeletal muscle has been associated to metabolic risk markers and insulin resistance in humans and mice (137, 138). However, little is known about the contribution of T cells infiltration to skeletal muscle inflammation and insulin resistance. Skeletal muscle T cells infiltration occurs in high-fat diet-fed mice (139). T cells localize within skeletal muscle in intermuscular and perimuscular adipose tissue suggesting that they might play a role in obesity-induced skeletal muscle inflammation and insulin resistance (13). Within skeletal muscle T cells polarized into pro-inflammatory INF-γ-secreting Th1-type inducing myocyte inflammation and insulin resistance through activation of JAK/STAT pathways, while Treg cells diminish in number. Interestingly, TCRb^−/−^ (TCR beta chain null) diet-induced obese mice show reduced skeletal muscle inflammation partially attributable to the lack of Th1 cells, confirming the role of T cells in skeletal muscle inflammation (139). Signals such as chemokines/cytokines/adhesion molecules that induce T cells infiltration into skeletal muscle are not yet identified. However, CD11a^−/−^ mice exhibited low inflammatory gene expression in VAT (140).

Administration of JAK1/JAK2 inhibitors *in vivo* reduces T cells infiltration within skeletal muscle and attenuates insulin resistance (13). Although there is no information, to our knowledge, about the presence of T cells infiltration in skeletal muscle in T1D, it has been described that a particular subpopulation of CD4^+^ T cells is associated with cachexia in NOD mice (141). In T2D, the level of transcriptome and proteome expression of activated T cells and muscle differ relative to non-diabetic controls (142). T cells, in particular Treg subsets, have homeostatic functions in muscle tissue repair regulating both the inflammatory response, by promoting the switching from M1 to M2 macrophages, and the activation of myogenic stem cells (143). However, further investigation will be required to choose any T lymphocyte subsets as potential targets for improving cachexia in diabetes.

## Hormones, Neuropeptides, and Neurotransmitters Modulate T Cell Function in Diabetes

T cell capacity to respond against foreign antigens while avoiding reactivity to self-peptides is mainly determined by cellular selection of developing T cells in the thymus (144). Positively selected cells migrate to the peripheral lymphoid organs and target tissues; however, extrathymic pathways of T cell differentiation have also been demonstrated to contributing to the generation of a wide functional spectrum of TCR repertoire.

Several hormones and neurotransmitters impact thymic microenvironment and peripheral tissues affecting T cell development in health and disease (6). In particular, numerous studies performed in human and mice models analyzed the neuroendocrine-immune systems relationship under the metabolic burden of diabetes.

### Growth Hormone (GH)—Insulin-Like Growth Factor-1 (IGF-1)

Growth hormone exerts pleiotropic functions modulating from carbohydrate, protein, and fat metabolism to the immune response (145). It is secreted by the anterior pituitary and also produced by immune tissues thereby acting in an autocrine/paracrine manner on immune cells (146).

It was reported that a single point mutation within the DNA binding domain of Stat5b, a central transcription factor downstream GH receptor, is a key molecular defect in NOD mice that limits Foxp3 expression in Treg cells (147, 148). Transgenic NOD mice overexpressing GH show normal glycemia throughout their lives; histochemical analysis of the pancreas revealed the development of peri-insulitis, but showed little or no islet infiltration or β-cell destruction (149). The authors demonstrated that this protective outcome involves several GH-mediated mechanisms on T cells, altering cytokine environment against a Th1 response, maintaining the activity of Treg cell subsets, and affecting Th17/Th1 plasticity. Additionally, sustained GH expression positively influenced β-cell viability.

Conversely, human studies reported that the incidence of T1D during GH replacement therapy in GH-deficient children was comparable with that of the general population (150–152) and described an association of GH treatment with disturbances on carbohydrate metabolism. The hyperglycemic effect of GH has been well-described mainly due to their action on liver, muscle, and adipose tissue (153–155). It is known that many of the GH effects are mediated by the production of IGF-1; thymocytes produce and release IGF-1 and also express its cognate receptor (156).

Several studies propose IGF-1 as a key factor able to induce protection from T1D. Human recombinant IGF-1 administration in NOD mice reduces the severity of insulitis and the incidence of autoimmune diabetes (157–159). The protective T cell-mediated effects of IGF-1 on T1D arose more recently. Anguela and colleagues showed that plasmid-delivered overexpression of IGF-1 in the liver prevents the development of hyperglycemia in a mice model of T1D; decreasing pancreatic infiltration, reducing apoptosis, and increasing replication of β-cell. In this experimental model, they observed an increase of intra-pancreatic Treg cell numbers and proposed an indirect effect mediated by IL-7-producing dendritic cells that improved Treg survival or by the conversion of conventional T cells into Tregs by TGF-β secreted from the liver (160). In a latter study, it was demonstrated that IGF-1 directly stimulates Treg cells proliferation *in vitro* in both mouse and human. Moreover, *in vivo* IGF-1 treatment *via* continuous delivery specifically stimulated proliferation of Treg but no other T cell subtypes and exerted protective action against autoimmune diabetes in two mice models [NOD and multiple low-dose streptozotocin (STZ) injections in C57BL/6J mice] (161). It is noteworthy that the protective effect of IGF-1 treatment might be also exerted at the β-cell level (162–164).

### Glucocorticoids (GCs)

Glucocorticoids are endogenous modulators of several biological processes including regulation of metabolism and stress response, and development of the immune system. In particular, GCs broadly affect T cell differentiation and function (165) with positive or negative effects depending on the dose at which they are exposed (166). Synthetic GCs are widely used for their immunosuppressive and anti-inflammatory properties to treat several immune disorders and preventing transplant rejection (167). Brief dexamethasone treatment during acute infection prevents virus-induced autoimmune diabetes in a rat model by down-modulating Th1 responses and restoring the balance between CD8^+^ T and Treg cells (168). However, the well-described severe side metabolic effects, such as osteoporosis, hypertension, and insulin resistance, induced by the chronic administration of GCs limits its therapeutic use for autoimmune diabetes (169). It is widely recognized the inhibitory action of GCs, when pharmacologically administered *in vivo*, on the proliferation of several human subpopulation of Ag- and mitogen-stimulated T cells (170). Mechanistically, the underlying inhibitory effects have been attributed to the ability of GCs to restrain gene expression of cytokines. In this respect, IL-2 has been indicated as the principal growth factor for T lymphocyte proliferation (171) However, under physiologic concentrations GCs show contrasting effects promoting TCR-stimulated T cell proliferation (172). CD4 acts as an important coreceptor during Ag recognition by the TCR, contributes to the assembly of TCR-MHC-II complex and thus, increases the sensitivity of T cell to the Ag presented by MHC-II lowering the amount of Ag required to mount an effective immune response. Corticosterone accelerates the expression of CD4 on T cell membrane (173). It has been reported that physiologic concentration of GCs regulates CD4 expression upon T lymphocyte challenge by Concavalin A or TCR stimulation. Also, CD8 expression is induced by GCs on activated mature T cells (174). Therefore, TCR triggering induces the expression of CD4 and CD8 on T lymphocytes and physiologic levels of GCs increase this process enhancing T cell activation.

Glucocorticoids affect gene expression by two main GR-dependent and -independent intracellular mechanisms that exert several biological effects. These differential mechanisms have fueled the interest in the study and development of new GR-ligands with dissociative properties combining GCs’ anti-inflammatory properties with a reduced side effect profile (175, 176). These particular dissociated GR-ligands hold potential for their use in Th1-mediated immune disorders. CpdA is a dissociating compound which does not stimulate GR response elements-driven gene expression (177). It has been reported that CpdA regulates T cells through inhibition of the master transcription factor T-bet and induction of GATA-3, thus inhibiting Th1 and favoring Th2 response (178).

In pregnant women at risk of preterm delivery, GCs are routinely administered in order to improve fetal lung development and newborn survival (179). The association of increased exposure to cortisol *in utero* (due to stress, pharmacological treatment, or impaired function of 11β-HSD-2) with long-term effects on glucose-insulin homeostasis has been demonstrated in human and animal models (180–183). However, studies regarding the effects of prenatal GCs on the development of autoimmunity are limited. Recently, using a mice model, Tolosa and colleagues demonstrated that prenatal administration of betamethasone increases apoptosis of developing thymocytes and induces changes in the TCR repertoire decreasing the frequency of pathogenic T cells and protecting from T1D development in NOD mice (184, 185). Conversely, an epidemiological study in Danish cohorts indicated the existence of an increased risk for T1D and T2D in young children who received prenatal steroid treatment (186). Under this scenario, a role of prenatal GCs exposure on pancreas development and T cell effects cannot be ruled out (187).

### Ghrelin and Leptin

Peptide hormones known to be involved in the control of eating behavior, glucose metabolism, and energy homeostasis, such as ghrelin and leptin, also exert regulatory effects on the immune system *via* their actions on several leukocytes, including T lymphocytes. Ghrelin and leptin are considered to play mutually antagonistic actions on food intake at the hypothalamic area (188, 189). The interplay between leptin and ghrelin at the level of immune cells was recently recognized. It seems likely in general terms that orexigenic peptides like ghrelin may play a role in promoting endogenous anti-inflammatory responses. On the other hand, anorexigenic agents like leptin might assist inflammation.

Ghrelin is mainly produced by endocrine-like cells in the stomach and released into peripheral blood. Also, the synthesis and secretion of ghrelin by T lymphocytes have been described (190). Human T lymphocytes constitutively express low levels of ghrelin which significantly increase upon cellular activation by stimulated TCR. Moreover, ghrelin enhances proliferation of peripheral CD4^+^ T cells and thymic murine T cells upon activation with anti-CD3/-CD8 mAbs and during its administration *in vivo*, respectively (191).

It was shown that ghrelin attenuated age-associated and GC-mediated thymic atrophy, and stimulated thymocyte proliferation in young and old mice *in vivo* through activation of its receptor GHS-R1a (191). Thymus involution with age correlates with lower expression levels of intrathymic ghrelin and its receptor, and exogenous administration of ghrelin partially reversed thymus involution and, consequent improvement of thymic progenitors and mature T lymphocytes (192). In addition, ghrelin action on suppressing inflammation might be attributed to the observed inhibition of T derived pro-inflammatory cytokines expression and Th17 development (190, 193). The acylated form of ghrelin exerts potent inhibitory effects on the expression of pro-inflammatory cytokines, such as IL-1β, TNF-α, and IL-6, as well as adhesion molecules by TCR-stimulated T cells. It has been suggested that these inhibitory actions of acylated ghrelin are mediated by GHS-R1a *via* specific blocking of NF-κB and/or STAT3 signaling (190).

There is also evidence that ghrelin is synthesized by T cells and inhibition of its production by using siRNA resulted in stimulation of INF-γ, IL-17 and other chemokines upon TCR ligation indicating that ghrelin might also influence T cell microenvironment regulating immune responses (193). Interestingly, ghrelin downregulates leptin-induced pro-inflammatory Th1 responses (190), suggesting that apart from counteract each other’s function at the level of energy homeostasis their interplay might influence T cells function as well. Ghrelin administration delays the development of autoimmune diabetes by reducing islet infiltration in BioBreeding rats; unfortunately, there is absence of information whether this hormone has any effect on diabetogenic T lymphocytes in this setting (194). However, it might be possible the regulation of diabetogenic T cell population through indirect mechanisms such as, an increase in the number or potency of Treg cells due to the reported modulatory effects of ghrelin on monocytes and dendritic cells (190, 195).

Leptin is an adipokine mainly secreted by white adipose tissue, which belongs to the family of the long-chain helical cytokines (IL-2, IL-15, and IL-12) commonly associated with pleiotropic functions. Leptin regulates feeding behavior and metabolism (196), hematopoiesis (197), angiogenesis (198), and reproduction (199). Also, leptin exerts modulatory actions on the immune systems (200). It was shown that leptin induces proliferation and secretion of IL-2 by CD4^+^ T lymphocytes in humans and mice (201). In addition, leptin assists Th1 cell-biased immune responses stimulating the secretion of INF-γ by T cells (202). Therefore, leptin promotes pro-inflammatory immune responses like the antigen-specific Th1-type directed against β cells observed in T1D. In fact, it has been reported that administration of leptin during early life accelerates the development of autoimmune diabetes in the NOD mice (203). Interestingly, Materese et al. found that circulating leptin peaked soon before the onset of hyperglycemia and spontaneous diabetes in female prone NOD mice. The administration of leptin enhanced the production of IFN-γ by peripheral T lymphocytes. On the other hand, a mutated version of the leptin-receptor in NOD mice suppresses autoimmune diabetes progression (204). All these evidences point leptin with its permissive action on the development of polarized Th1-type autoimmunity against β cells.

### Insulin

Only sparse data are available regarding the role of insulin on T lymphocytes. It has been reported that insulin infusion resulted in reduction of NF-κB and ROS generation, and increase in IκB in mononuclear cells, all changes characteristic of an anti-inflammatory effect at the molecular level (205). Unfortunately, this study did not address whether there is a similar response to insulin treatment in all mononuclear cells or there is a particular cellular type more sensible to insulin action. Later, it was elucidated that insulin drives T cell differentiation toward an anti-inflammatory Th2-phenotype by mechanisms that involve ERK activation (206). Nevertheless, other study found that in T cells isolated from obese subjects incubation with supra-physiological concentration of insulin did not alter the Th1/Th2 balance suggesting that insulin signaling in lymphocytes is strongly impaired in obesity, shifting T-cell differentiation toward a pro-inflammatory phenotype (207). During diabetes there is a high occurrence of apoptosis in lymphocytes and insulin treatment reduces this effect, suggesting that insulin may act as a pro-survival factor for lymphocytes (208). Moreover, there is evidence in favor of a role of insulin in promoting obesity-associated adipose tissue inflammation (209).

A recent theoretical study simulated how hyperinsulinemia might alter the dynamics of the CD4^+^ T regulatory network (210); the analysis showed how high insulin levels affect the differentiation and plasticity of CD4^+^ T cells favoring stabilization of inflammatory Th1 and Th17 and reducing the stability of Treg types. In line with this *in silico* observations, it has been demonstrated *in vitro* that Tregs express the insulin-receptor and that high levels of insulin specifically inhibits IL-10 production *via* AKT/mTOR signaling and impairs the ability of Treg cells to suppress TNF-α production by macrophages (211). Moreover, the authors showed that Tregs from the VAT of hyperinsulinemic diet-induced obese mice exhibited a specific decrease in IL-10 production, as well as a parallel increase in IFN-γ production; suggesting that hyperinsulinemia may contribute to the development of obesity-associated inflammation *via* modulation of Treg function.

Resting T lymphocytes do not express detectable levels of insulin-receptor; however, after activation its expression is significantly increased (206, 212, 213). A more recent study suggests that upregulation of the insulin-receptor on activated T cells is critical for T cell function and efficient adaptive immune response (214). In conditions of impaired insulin-receptor expression, T-effector activities are diminished resulting in attenuated clinical symptoms in a T-cell-mediated multiple sclerosis model *in vivo* (214). Fischer et al. showed that silencing the insulin-receptor on T lymphocytes disrupts their function, such as reducing cytokine production, proliferation, and migration without affecting thymocytes development. Interestingly, the absence of insulin-receptor affected CD4^+^ and CD8^+^ T subsets whereas the frequency and potency of Treg cells were unaffected (214).

T lymphocytes use aerobic glycolysis (Warburg effect) upon activation and their increase in glucose demand is facilitated by induction of the insulin-receptor along with GLUT1 (215). Given the critical dependence on glucose upon activation, glycemic status should be considered as a factor affecting T cell function. The diabetic state, where circulating glucose levels are elevated, provides an environment of oxidative stress and activation of the inflammatory pathways. Transgenic expression of Glut1 augmented T cell activation and led to accumulation of readily activated memory-phenotype T cells with signs of autoimmunity in aged mice (216). Increased glucose uptake may lead to excessive T cell activity and accumulation as a result of enhanced T cell activation and/or inhibition of T cell death following stimulation. Moreover, human CD4^+^ and CD8^+^ T cells differ in the relative use of the metabolic pathways contributing to functional responses. Thus, CD4^+^ T subset shows higher basal glycolysis mainly attributed to elevated expression of glycolytic enzymes and CD8^+^ T subpopulation showing a decrease in glycolysis upon activation and greater dependency on mitochondrial metabolism for cytokine production. Also, it was demonstrated that the binding affinity of specific antigens fine-tune T cell metabolism (217). Therefore, T lymphocyte insulin-receptor/GLUTs expression, insulin and glucose levels as well as, the affinity of antigens with cognate TCR of different T cell subsets all have implications to consider for therapeutic manipulation in the setting of hyperglycemia and hyperinsulinemia (T2D) and, during T-cell-mediated T1D featured by elevated glycemia and lack of insufficient insulin levels.

### Prolactin (PRL)

Prolactin is a pituitary hormone not only essential for reproduction and lactation but also involved in immunological responses. PRL and its receptor are expressed by various extra-pituitary tissues, including lymphoid cells (218, 219). PRL has a stimulatory action on the immune system; it affects differentiation and maturation of both, B and T lymphocytes, stimulates lymphocyte proliferation and macrophage function, and enhances inflammatory responses and production of immunoglobulins (220–222).

Increase serum PRL has been detected in autoimmune disorders including T1D and elevated prolactinemia was also observed in T2D (223–225). The association between circulating PRL levels and glucose homeostasis has been controversial. Within the physiological range, higher serum PRL levels seem to be associated with insulin resistance in men (226) and with reduced glucose tolerance in the third trimester of pregnancy in women (227). Conversely, higher circulating PRL levels were associated with lower prevalence of diabetes and impaired glucose regulation in a large cohort of middle-aged and elderly men and postmenopausal women (228).

Experimental studies suggested a protective role associated with PRL modulation of T cell development; PRL reduces insulitis and protects against autoimmune diabetes in NOD mice (229) in the autoinmune diabetes model induced by low-dose STZ administration in C57BL/6 mice (230). Further studies in this latter experimental model showed that PRL treatment enhances a Th2 response by increasing the frequency of IL-10 positive splenocytes and down-modulating the featured expression of the Th1 cytokines IFN-γ and TNF-α in splenocytes (231). Furthermore, PRL-expanded Treg (CD4^+^ Foxp3^+^) population and improved the efficacy of short-term low-dose anti-CD3 treatment (which induce a transient CD4^+^ and CD8^+^ T cell depletion) at achieving diabetes remission in the NOD mice (232). Conversely, severe hyperprolactinemia induced by anterior pituitary ectopic transplantation increases the incidence of diabetes in the NOD mice (233). A study analyzing the *in vitro* effect of PRL on CD4^+^ T cell suggested that the modulatory effect is dose dependent; low-dose PRL promotes Th1 response through increases in its Th1-driven transcription factor T-bet, whereas higher doses have suppressive effects (234). Therefore, differences obtained in clinical and experimental studies might be explained on the basis of the PRL differential effect on T cells, glucose metabolism, and insulin resistance depending of the hormone concentration impacting on target tissues.

Moreover, it was demonstrated that PRL stimulates insulin secretion and proliferation of β-cells in murine and human islets (235–237) and in particular during pregnancy (238). Thus, a further protective action of PRL exerted at β-cells level could not be ruled out in the experimental models studied.

### Oxytocin (OXT)

Oxytocin is an essential neuropeptide involved in the regulation of maternal behavior, lactation, and parturition (239). In the central nervous system OXT is expressed in subpopulations of hypothalamic neurons, stored in the neurohypophysis and released into circulation. Besides its central origin, OXT is produced and released in peripheral tissues acting in a paracrine and autocrine fashion *via* widely expressed OXT receptors (240). In addition to the abovementioned physiological functions in mammals, the modulatory effect of the OXT-secreting system on immune system activity and metabolic homeostasis has come to gain attention.

Oxytocin effects on immune functions include thymus physiology, immunologic defense, homeostasis, and surveillance (241). However, scarce information exists regarding the interaction of OXT with T lymphocytes in diabetes. CD38, a membrane ADP-ribosyl cyclase expressed in several cells such as lymphocytes and β-cells, is involved in OXT secretion (242); targeted disruption of CD38 accelerates autoimmune diabetes in NOD mice by enhancing autoimmunity (243). CD38-deficient mice presented a disbalance between T-effector and Treg cells and an age-dependent increase in a diabetogenic CD8 clonotype, along with impaired insulin secretion and an elevated plasma glucose level.

Recent studies have shown that the impairment of OXT signaling is associated with disturbance of metabolic homeostasis, resulting in obesity and diabetes. In mice under a high-fat diet, there was a significant increase in both OXT and OXT receptor levels in the brain, as well as an increase in OXT receptor in the islets (244). OXT receptor-deficient mice exhibited increase β-cell death under metabolic stress conditions resulting in impaired insulin secretion and glucose intolerance under a high-fat diet (244). Both OXT- and OXT receptor-deficient mice developed late-onset obesity (245, 246).

On the other hand, peripheral OXT treatment improved glucose tolerance and reduced food intake and visceral fat mass in mice under diet-induced obesity (247, 248). Moreover, OXT treatment improved glucose homeostasis and induced tissue regenerative changes of pancreatic islets after STZ-induced diabetes in rats (249); similar results were obtained in mice (248). Conversely, worsening of basal glycemia and glucose tolerance were observed under OXT treatment in ob/ob animals (250) suggesting that OXT effects on glucose metabolism may depend on the interaction with leptin signaling.

A central action of OXT on glucose homeostasis was also observed. Intranasal OXT delivery enhanced glucose tolerance and β-cell response in healthy men challenged with an oral glucose tolerance test (251). Furthermore, OXT nasal spray treatment in obese patients effectively reversed obesity and related lipid disorders and improved blood glucose and insulin postprandial levels (248). In addition, third-ventricle injections of OXT improved glucose intolerance and fasting blood insulin levels in mice under chronic high-fat diet feeding and led to significant improvements in glucose tolerance, β-cell insulin secretion, and blood insulin levels in the multiple low-doses administration of STZ-induced autoimmune diabetes in mice (248).

### Sexual Steroids

For most of autoimmune diseases, females are generally more frequently affected than males. This is the case for systemic lupus erythematosus, rheumatoid arthritis, and multiple sclerosis. However, sexual dimorphism in autoimmune diabetes prevalence is observed in NOD mice but not in humans (252). One of the main factors contributing to gender differences in immune system is sex hormones. The effects exerted by female (estrogen, progesterone) and male (androgens) steroid hormones on T lymphocytes might explain gender differences in specific autoimmune diseases (253).

Several studies indicate that testosterone has suppressive effects on T cells by inhibiting Th1 differentiation of naive CD4^+^ T cells and pro-inflammatory cytokine production and enhancing the expression of anti-inflammatory cytokines (254, 255). Ovarian hormones also modulate T lymphocyte function. *In vivo* and *in vitro* evidence indicate that progesterone, which promotes maternal–fetal tolerance during pregnancy, favors the Th2, and suppresses Th1 and Th17 responses, and has a potent Treg induction activity promoting the production of anti-inflammatory cytokines like TGF-β1 and IL-10 (256, 257). Numerous evidences support estrogens influence on the development and maintenance of thymic and peripheral T cell function with dual effects depending on factors, such as steroid concentration, target cell, and timing (258). Estradiol at periovulatory to pregnancy levels stimulates IL-4 and IL-10 production and inhibits TNF-α from CD4^+^ T cells and increases Th2 and Treg phenotype, which might shift the immune response toward tolerance (258, 259). On the other hand, at lower concentrations, estradiol stimulates TNF-α, IFN-γ, and IL-1β production (258, 260). Scarce information is available regarding sexual steroids and T cell interaction under the burden of diabetes.

NOD mice spontaneously develop diabetes with a strong female prevalence; a more invasive and destructive insulitis, leading to an earlier onset and higher incidence is observed in females (261). Moreover, the incidence of diabetes was significantly decreased in female NOD mice, but increased in male, by castration at the time of weaning (262, 263). Furthermore, long-term administration of androgen or its derivatives to young female NOD mice resulted in a decrease in the percentage of CD4^+^ T cells in peripheral blood mononuclear cells and the incidence of diabetes (264, 265). Bao and colleagues demonstrated that sex hormones modulate the Th1/Th2 balance in the early stages of the T cell-mediated autoimmune process in the NOD mice; IFN-γ expression was significantly higher in pancreatic and lymph node-T cells from young females, whereas IL-4 expression was higher in male counterparts. This differential expression, enhancing Th1 immune response in female NOD mice, was found to be due to the upregulation of IL-12 induced IFN-γ production through activation of STAT4 by estrogen (266). Additionally, it was suggested that male-specific gut microbiome play a protective role in NOD mice that is mediated, at least in part, *via* microbiota metabolism of sex hormones (267). Conversely, estradiol administration was found to restore immunomodulatory functions of iNKT cells and preserve female NOD mice from both spontaneous and cyclophosphamide-induced diabetes (268).

A clear sexual dimorphism is observed related to glucose metabolism and obesity-associated T2D. The sex difference in the prevalence of diabetes was reversed during reproductive life, there are more men with T2D at middle age while there are more affected women after menopause (269), suggesting a protective role of estrogens. Consistent with this observation, continuous estradiol treatment (pregnancy levels) in males inhibited weight gain and the associated onset of hyperglycemia in an islet amyloid (huIAPP)-dependent murine model of diabetes; histological analysis of the pancreas revealed estradiol prevented deposition of islet amyloid and preserved islet mass and β-cells insulin content (270). Mice of both sexes develop a vulnerability to STZ-induced insulin deficiency when estradiol production or signaling is genetically suppressed (aromatase-deficient, ArKO^−/−^ and ERα-deficient, ERKO^−/−^ mice); in these mice, estradiol treatment prevents STZ-induced β-cell death and helps sustain insulin production, and prevents diabetes (271). Estradiol protective effect on β-cells was also observed in isolated human pancreatic islets; estradiol treatment of cytokine-challenged islets increases islet viability by lowering NF-κB activity and caspase-9 activation and cytokine-induced cell death. Additionally, estradiol improved glucose-stimulated insulin response *in vitro* and *in vivo* functionality of treated human islets after transplantation in the portal vein of STZ-induced NOD*scid* mice (272).

Estrogen protective action on glucose homeostasis is not only exerted in the pancreas; several studies indicated that estradiol enhances insulin sensitivity in peripheral tissues, improves body fat distribution, and reduces adipose tissue inflammation (273–275). Estrogen treatment prevented insulin insensitivity and reduced the expression of adipose tissue inflammation (*Mcp-1* and *Cd68*) induced by high-fat diet in ovariectomized mice (274).

Although its protective anti-inflammatory effect on immune cells, progesterone has been associated with the development of gestational diabetes. It was demonstrated that the hyperglycemic effect of gestational levels of progesterone is mostly due to the enhancement of insulin resistance (276), particularly by a reduction of glucose transporter 4 expression in skeletal muscle and adipose tissue (277) but also reducing insulin secretion by a non-genomic mechanism (278). A recent study performed in RINm5F β-cell line and primary rat islets show that progesterone, particularly at pharmacological concentrations used for preterm delivery prevention, induced apoptosis of pancreatic β-cells through an oxidative-stress-dependent mechanism (279), contributing to gestational diabetes pathogenesis.

It is well established the impact of testosterone deficiency on the development of visceral obesity and insulin resistance in men (280, 281). Consistently, androgen receptor-deficient mice exacerbates adiposity and insulin resistance induced by a high-fat diet; elevated serum IL-1β levels and decreased pancreatic glucose-stimulated insulin secretion was also observed (282). A recent transcriptome analysis of islets from adult male mice lacking androgen receptor selectively in β-cells revealed alterations in genes involved in inflammation and β-cell function (283).

Recently, Rubinow and colleagues analyzed lymphocyte subsets in subcutaneous adipose tissue biopsies after 4 weeks of pharmacological testosterone suppression with a GnRH receptor antagonist and controlled testosterone replacement in healthy male subjects. In this clinical study, change in serum total testosterone levels correlated inversely with CD3^+^, CD4^+^, and CD8^+^ T cells and ATMs within adipose tissue (275).

At the pancreas level, it was observed a sex specific protective action of testosterone on STZ-induced apoptosis in β-cells; the cytoprotective effect was seen in gonadectomized male but not in female rats (284, 285). Moreover, chronic hyperandrogenism induced β-cell dysfunction and failure to compensate high-fat diet induce insulin resistance in female mice (286). The sexual dimorphism in the modulation of glucose and energy homeostasis by testosterone is evidenced in the clinic, androgen excess predisposes to insulin resistance, β-cell dysfunction, and T2D in women (281). Nonetheless, further research is needed to reveal the mechanisms underlying the sex differences in the metabolic effect of testosterone.

### Neurotransmitters

Originally, the notion that neurotransmitters act as immunomodulators emerged with the discovery that their release from the nervous system could lead to signaling through lymphocyte cell-surface receptors modulating immune response. It is now known that neurotransmitters can also be released from immune cells and act as autocrine or paracrine modulators.

It has been demonstrated that administration of gamma-aminobutyric acid (GABA), a major CNS neurotransmitter synthesized from glutamate by glutamic acid decarboxylase (GAD), exerts antidiabetic effects by acting on both islet β-cells and the immune system in both T1D and T2D models. GABA acts as an autocrine excitatory neurotransmitter in human pancreatic β-cells through GABA receptors (287, 288).

Gamma-aminobutyric acid promotes proliferation, protects β-cells from STZ- and cytokine-induced apoptosis (288), and inhibits human β-cell apoptosis following islet transplantation into NOD*scid* mice (289). This protective effect is also observed *in vivo*, e.g., GABA treatment prevents insulitis and diabetes onset and preserves insulin expression in NOD mice and in multiple low-dose STZ-induced diabetes in C57BL/6 mice (288, 290, 291) and delays hyperglycemia in the adoptive transfer of disease in NODscid mice (292). Moreover, overtly diabetic NOD mice treated with GABA improved fasting glycemia, insulin and C-peptide levels and glucose tolerance (291).

Also, GABA receptors are expressed in various immune cells, including T cells (292, 293). Low doses of GABA inhibited activated T cell responses against islet autoantigens when assayed *ex vivo* (292), suggesting that GABA downregulates diabetogenic Teff function *in vivo*. Later studies showed an anti-inflammatory effect of GABA treatment, increasing the frequency and suppressive activity of splenic CD4^+^Foxp3^+^ Tregs in pancreatic lymph nodes in NOD mice with no changes in GAD-reactive CD4^+^ T cells and decreased circulating inflammatory cytokines in the multiple low-dose STZ-induced diabetes model (288, 291).

A beneficial effect of GABA was observed also in T2D experimental models. Oral GABA administration inhibited obesity, reduced fasting blood glucose, and improved glucose tolerance and insulin sensitivity in high-fat diet-fed C57BL/6 mice. Moreover, even after the onset of obesity and hyperglycemia, GABA treatment improved glucose homeostasis (294). Furthermore, GABA treatment inhibited obesity-related inflammation, reducing the frequency of VAT macrophage infiltrates and increasing the frequency of splenic CD4^+^Foxp3^+^ Tregs in high-fat diet-fed mice (294).

In accordance with the antidiabetic effect in preclinical models, GABA and GABA analogs were also shown to exert insulinotropic effects in humans (295, 296).

Interestingly, consistent with the high levels of GAD found in the islets of Langerhans, GAD65 is one of the major target autoantigens recognized by self-reactive T cells in T1D. Complete suppression of β-cell GAD expression in NOD mice blocked the generation of diabetogenic T cells, protected islet grafts from autoimmune injury and consequently, the development of autoimmune diabetes (297). In fact, potential immunomodulation with GAD therapy has been extensively investigated for the prevention or treatment of T1D in humans (298).

Histamine is an inflammatory mediator classically involved in allergic reactions but also in the modulation of innate immunity and autoimmune reactions. Its diverse effects are mediated by the differential expression and regulation of four known histamine receptors (termed H1R-H4R) and their distinct intracellular signals (299). Th1 and Treg cells express relatively high levels of H1R, whereas H2R is preferentially expressed by Th2 cells. Histamine modulates T lymphocytes by enhancing Th1 responses through H1R and downregulates both the 1- and 2-type responses through H2R (300); activation of H1R by histamine decreases Treg cell suppressive functions.

The association of autoimmune diseases, such as multiple sclerosis, rheumatoid arthritis, and diabetes, and elevated serum and tissue histamine levels was described many years ago (301–303). However, research searching for the possible role of histamine signaling on diabetes emerged recently.

In histidine decarboxylase (HDC) deficient NOD mice, the lack of endogenous histamine reduces IL-12 and IFN-γ levels and delays the onset of autoimmune diabetes (304); the proportion CD4^+^CD25^+^Foxp3^+^ Treg cells in spleen and pancreatic lymph node remained unchanged. Surprisingly, exogenous histamine administration not only failed to increase the incidence of T1D but also delayed the onset of disease in both wild-type and HDC^−/−^ mice (304).

Central histamine signaling is involved in the control of feeding behavior and energy homeostasis. H3R is principally expressed in histamine neurons and negatively regulates the synthesis and release of histamine. Treatment with a H3R agonist decreases appetite, body weight, and insulin resistance in diet-induced obese mice (305). On the other hand, targeted disruption of H3Rs leads to an obese phenotype (306). Moreover, mice deficient in histamine H1R or HDC showed a dysregulation in the leptin signaling, impaired glucose tolerance, and are prone to become obese on a high-fat diet or at advanced age (307–309).

It was recently reported that the H1R antagonist cetirizine partially counteracts cytokine- and oxidative stress-induced β-cell death (310). *In vivo*, H1R antagonist ameliorates high-fat diet-induced glucose intolerance in male C57BL/6 mice, but no effect was observed on diabetes outcome in female NOD mice, suggesting a protective effect of cetirizine against high-fat diet-induced β-cell dysfunction, but not against autoimmune β-cell destruction (311).

## Conclusion

**Figure 1:**
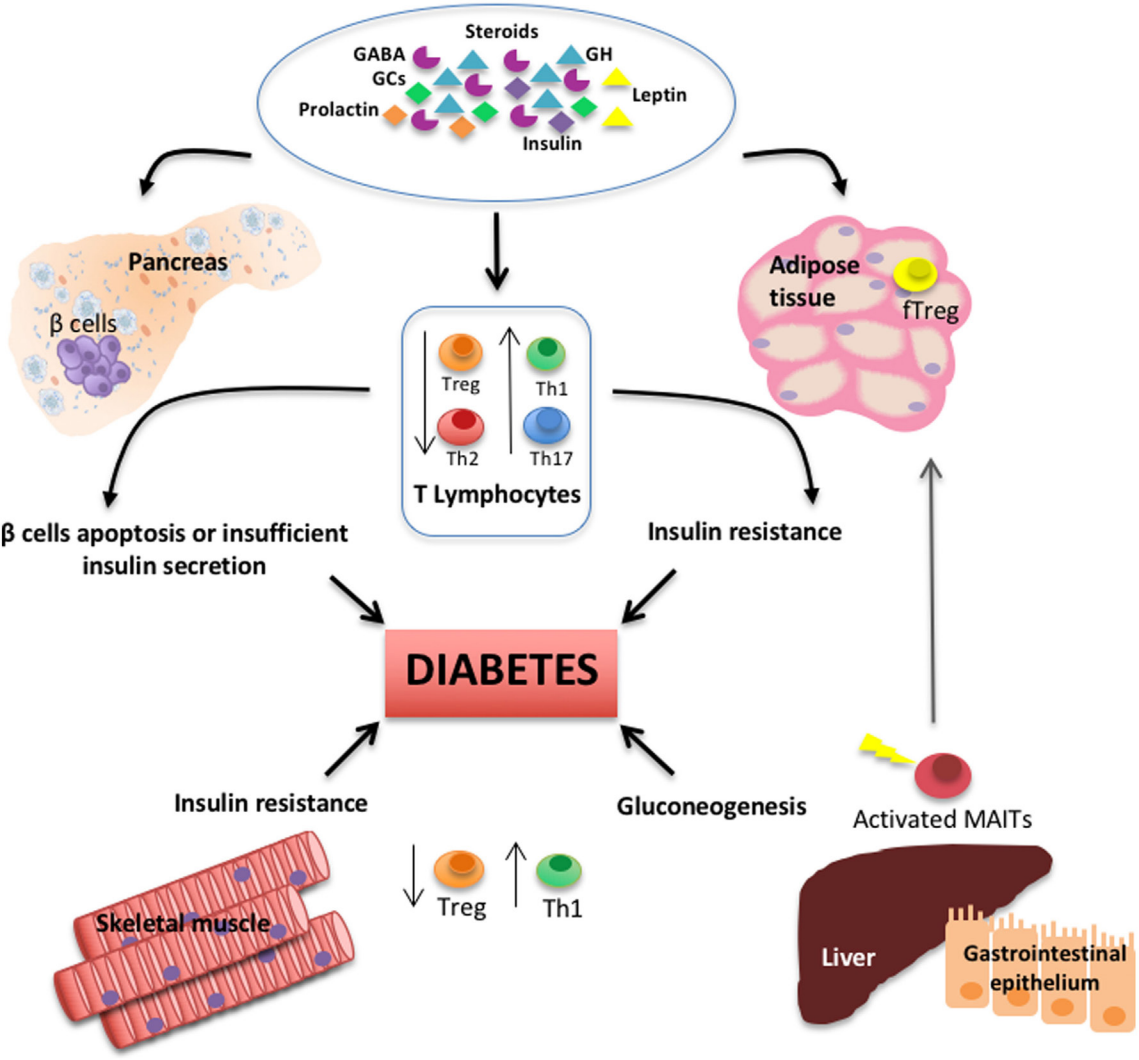
Schematic diagram depicts the interplay among T lymphocyte subsets, several types of hormones and neurotransmitters, and primary peripheral tissues regulating glucose metabolism and during the pathogenesis of diabetes.

T lymphocytes, as important components of the adaptive arm of the immune system, are key players in the modulation of metabolism in several tissues in health and disease (see Figure 1). The neuroendocrine system plays an essential role controlling the number and activity of different T cell subpopulations. Herein, we collected data that warrant further investigation on T lymphocytes biology hoping that it would lay the groundwork for future translational research that aims to restore homeostasis in metabolic disorders and treat diabetes in its multiple forms.

## Author Contributions

LA and MP contributed to the conception and design of the review article and wrote sections of the manuscript; MG created the model figure. All authors contributed to manuscript revision, read and approved the submitted version.

## Conflict of Interest Statement

The authors declare that the research was conducted in the absence of any commercial or financial relationships that could be construed as a potential conflict of interest.
